# Status and prospects of percutaneous vertebroplasty combined with ^125^I seed implantation for the treatment of spinal metastases

**DOI:** 10.1186/s12957-015-0484-y

**Published:** 2015-03-25

**Authors:** Lin Xie, Yanjin Chen, Ya Zhang, Zuozhang Yang, Zhaoxin Zhang, Lida Shen, Zhongqin Yuan, Mingyan Ren

**Affiliations:** Department of Medical Oncology, Bone and Soft Tissue Tumours Research Centre of Yunnan Province, Third Affiliated Hospital of Kunming Medical University, Tumour Hospital of Yunnan Province, Kunming, Yunnan 650118 PR China

**Keywords:** ^125^I Seed implantation, Bone cement, Metastatic tumour, Percutaneous vertebroplasty, Spine

## Abstract

Metastatic spinal tumours are the most common type of bone metastasis. Various methods have been used to treat metastatic spinal lesions, including radiotherapy, chemotherapy, isotope therapy, bisphosphonate therapy, analgesics, and surgery. Conservative treatments such as radiotherapy and chemotherapy are not appropriate and usually are ineffective in patients with vertebral fractures and/or spinal instability. Minimally invasive surgical treatments using non-vascular interventional technology, such as percutaneous vertebroplasty (PVP), have been successfully performed in the clinical setting. PVP is a non-invasive procedure that creates small wounds and is usually associated with only minor complications. In the present study, we will review the clinical status and prospects for the use PVP combined with ^125^I seed implantation (PVPI) to treat spinal metastases. The scientific evidence for this treatment, including safety, efficacy, and outcome measures, as well as comparisons with other therapies, was analysed in detail. PVPI effectively alleviates pain in metastatic spinal tumour patients, and the use of interstitial ^125^I seed implants can enhance the clinical outcomes. In conclusion, PVPI is a safe, reliable, effective, and minimally invasive treatment. The techniques of PVP and ^125^I seed implantation complement each other and strengthen the treatment’s effect, presenting a new alternative treatment for spinal metastases with potentially wide application.

## Review

Metastatic spinal tumours are the most common type of bone metastasis, and 39% of bone metastases are located in the axial skeleton [1,2]. The thoracic spine is the most common site of involvement, followed by the lumbar spine and the cervical spine [3]. Spinal metastases are often seen in patients with breast, lung, and prostate cancers. The majority of spinal metastases are due to haematogenous spread; a small number of lymphatic metastases are also seen. The vertebral body is much more easily damaged by spinal metastases than its the other parts of the vertebra due to its large volume and the abundance of blood vessels within it. Many tumour cells in spinal metastases produce and secrete osteoclast-stimulating factors, thus enhancing bone absorption and leading to bone destruction. Metastatic lesions can ultimately destroy vertebral bodies and their attachments, resulting in spinal deformity and spinal instability [4], which can cause severe back pain and neurologic dysfunction. The quality of life and psychological state of patients with spinal metastases may be seriously affected.

Various treatment methods, including radiotherapy, chemotherapy, isotope therapy, bisphosphonate therapy, analgesics, and surgery, have been used to treat metastatic spinal lesions. Conservative treatments such as radiotherapy and chemotherapy are not appropriate and usually are ineffective in patients with vertebral fractures and/or spinal instability. Open surgery often requires a significant postoperative recovery period and can prevent or delay treatment of the primary tumour. Indeed, open surgery can lead to increased mortality in patients with primary tumours. In addition, it is often more difficult to treat patients with non-adjacent and multi-segmental vertebral metastases with open surgery.

Recently, minimally invasive surgical treatment with percutaneous vertebroplasty (PVP), which is a non-vascular interventional technology, has been successfully performed in the clinical setting. PVP is a minimally invasive, image-guided therapy in which bone cement is injected at sites of vertebral lesions by percutaneous puncture, thereby enhancing the strength of the vertebral bodies and improving spinal stability. PVP is a well-established local anti-cancer treatment that can be used to relieve back pain from a vertebral body fracture.

Interstitial radiation therapy with ^125^I seed implantation is a new after-loading technology that has been increasingly used in recent years. It is a new, effective, and minimally invasive therapy in which encapsulated radioactive particles with certain specifications and activity are directly implanted into tumour tissues to provide low-dose, long-term radiation exposure for therapeutic purposes. Currently, interstitial radiation therapy is used to treat multiple solid tumours, and it has been widely shown to effectively inhibit tumour growth, relieve pain and improve patients’ quality of life.

Yang et al. [5] reported that the combined technique of PVP and ^125^I seed implantation (PVPI) into the vertebral body for bone interstitial brachytherapy in the treatment of spinal metastases produced better clinical results. PVPI is a novel composite treatment technology that combines two types of treatment and has a unique technological advantage.

Herein, we review the clinical status and application prospects of this new method in the treatment of spinal metastases by PVP combined with ^125^I seed implantation (PVPI).

## Status of the clinical use of PVP

PVP was developed as a method to inject bone cement into lesions of the vertebral body by percutaneous puncture. It enhances the strength of the vertebral body and the stability of the spine, thus preventing collapse, relieving back pain and backache, and allowing partial restoration of vertebral height. The French radiologists Galibert et al. [6] successfully treated a patient with chronic pain caused by a C2 vertebral haemangioma with PVP for the first time in 1984. In 1989, Kaemmerlen [7] used the technology in the treatment of patients with vertebral body metastases. Thus, PVP has been used for nearly 20 years. During this time, its use in the treatment of spinal metastases has gradually been extended worldwide, and its beneficial effects have been extensively recognized by clinicians and patients [8-10].

### Indications for PVP

PVP is primarily used in three specific clinical situations. The first is in cases of vertebral compression fractures, which often occur in older patients and are usually treated with bed rest and medication. However, bed rest usually aggravates osteoporosis and can easily lead to re-compression, resulting in a vicious cycle. PVP can rapidly relieve back pain caused by osteoporosis, stabilize the spine, and prevent vertebral collapse and fracture recurrence. Second, PVP is often used to treat vertebral haemangiomas, which are usually asymptomatic, benign lesions. When pain occurs, radiotherapy has historically been the major treatment method despite the variety of possible complications. Hence, radiotherapy has gradually been replaced by PVP [11]. Gilbert et al. [12] believed that 80% of patients thought they were effectively treated by PVP after a short period of time, and 73% of patients thought it had a significant analgesic effect and improved their quality of life. In a third clinical application, PVP is used to carry certain drugs, such as bone cement mixed with bone growth hormones, other biologically active substances, or anticancer drugs; the resulting effects have been shown to be better than those obtained with PVP alone.

### Selection and application of filler material

The following filling materials are commonly used for PVP in the clinical setting:

- Polymethylmethacrylate (PMMA) bone cement does not have bone-inducing activity or conductivity; it requires high temperature for polymerization.
- Carbonated hydroxyapatite bone cement has bone-conducting properties and good tissue compatibility. It does not produce heat and integrates with new bone during the curing process.
- Calcium phosphate cement is a novel bone substitute that has bone-conducting properties and is biocompatible. It can restore the height and biomechanical strength of vertebral bodies without causing significant inflammatory effects. Later, mechanical fixation can be changed to biological fixation.
- Ceramic materials that have biological activity and opacity can be chemically combined with the bone cement. In this way, the elastic modulus of the bone cement can be improved and the hardness of the vertebral body increased.

### Treatment of spinal metastases with PVP

PMMA is inexpensive and widely used in clinical orthopaedics; it is the most commonly used substance for PVP, leading to the accumulation of extensive experience with this material. Cold storage and the use of a suitable ratio of powder to liquid to contrast agent can lengthen the intraoperative injection window and improve the ability of the material to set [13,14]. A study to identify the optimal proportion of materials and temperature for locally available cement demonstrated that the most suitable material for PVP is domestic PMMA in toothpaste form that has been kept at 4°C, with a powder (g): liquid (mL): contrast agent (mL) ratio of 3:2:1 at room temperature [15].

The other filling agents mentioned above, with their good bone conductivity, are more appropriate for vertebral bone fusion and thus are suitable for the treatment of osteoporotic compression fractures. We believe that when spinal tumours are treated by PVP, the residual tumour tissues are mainly and inevitably distributed in the periphery of the bone cement core, between the nuclear area of the bone and the normal bone cement. Because these distribution characteristics suggest that a filling agent with bone-conducting properties has only limited advantages, the above material is not suitable for PVP treatment of spinal tumours.

### PMMA filling dosage and distribution

Cotton et al. [16] reported that the required amounts of vertebral bone cement filling were, on average, 2.5 mL in the cervical spine, 5.5 mL in the thoracic spine, and 7.0 mL in the lumbar spine. Vertebral body stiffness recovery is closely related to the quantity of bone cement used for filling. If the bone cement fills 14% of the vertebral body, recovery will proceed to the same level as was present before damage occurred. If 30% of the vertebral body is filled, more than half of the initial shear stiffness will be lost. Under the same loading conditions, measurements of unilateral pedicle puncture and bilateral stiffness are similar. Therefore, excessive filling does not produce optimal biomechanical effects, and a reasonable treatment strategy for the vertebral body includes the use of a small, symmetrically distributed amount of bone cement filling [17,18]. The use of excessive bone cement filling material increases the likelihood that surgery will be necessary due to leakage of bone cement and the occurrence of bone cement implantation syndrome.

## Clinical applications of ^125^I seeds

Interstitial brachytherapy with radioactive particles has been used to treat tumours for more than 100 years. Roentgen discovered ^125^I radiation in 1895. In 1901, Pierre Curie was the first to propose interstitial brachytherapy, which involves embedding radioactive sources directly within cancerous tissue. In 1903, Strebel treated tumours by inserting radium-226 into the tumour through a needle, thus creating a precedent for the treatment of tumours by interstitial implantation with radioactive sources.

In brachytherapy, the distance from the radioactive source to the target site should be less than 5 cm, which is different than the optimal distance for conventional external beam radiotherapy [19]. Due to the unique physical properties of radionuclides, high doses of radiation are produced in the target area. However, the dose is rapidly attenuated in the normal tissues surrounding the tumour. Thus, interstitial brachytherapy can kill tumour cells, protect normal tissues, and reduce complications [20].

Available radionuclides for interstitial radiotherapy include ^60^Co, ^137^Cs, ^182^Ta, ^191^Ir, ^198^Au, ^131^Cs, ^125^I, and ^103^Pb; of these, ^125^I and ^103^Pb are the most commonly used. The biological effect of ^103^Pb is higher than that of ^125^I (initial dose rate was 7 cGy/h, ^125^I was 7.5 cGy/h). The energy emitted by these radioisotopes can effectively kill cancer cells, while adjacent tissues and organs are not damaged. Compared with ^103^Pb, the advantages of ^125^I are its low energy, long half-life, and suitability for use in slow-growing tumours. ^125^I is the most widely used material for permanent implantation.

^125^I, an artificial isotope, was first produced in 1965. ^124^Te absorbs a neutron and is converted into ^125^I by electron capture, and ^125^I spontaneously decays into the excited state of ^125^Te by electron capture. In total, 93% of the decay energy is transformed into X-rays and electron beam through the process of decay; 7% of the decay energy is released as γ -rays with an energy of 35.5 KeV, which mainly interact with tissues via the photoelectric effect.

In 1965, Whitmore successfully treated prostate cancer at Memorial Hospital in New York with the first clinical application of ^125^I seeds. In 1974, the first permanent implant treatment of radioactive ^125^I particles to cure non-resectable and malignant tumours, effectively prolonging a patient’s life, was performed at Stanford University Hospital. In 1986, Theragenics began producing implantable particles of ^125^I and ^103^Pb that were certified by the United States FDA; permanent implant therapy thereafter quickly came into wide use and has been extensively studied. Currently, interstitial brachytherapy is routinely used to treat many tumours and has become a standard treatment for early-stage prostate cancer in the United States. Worldwide, reports have discussed the use of ^125^I particles for the treatment of brain tumours, solid tumours (including lung, liver, pancreatic, cervical, and other tumours), and metastatic spinal cancer, among other malignancies.

### Structure of ^125^I particles

The ^125^I capsule for brachytherapy applications features a titanium pipe with a diameter of 0.8 mm, a length of 4.5 mm, and a wall thickness of 0.05 mm; at its centre is a silver bar, 0.05 mm in diameter and 3.0 mm in length, which is permeable to the radiation emitted by the radioactive ^125^I nuclide. ^125^I has a half-life of 59.41 days, and its electron capture decay process yields a characteristic X-ray and an internal conversion electron, the titanium wall can absorb the ^125^I particle source. The main Te-KX characteristic X-rays are 27.4-KeV, 31.4-KeV, and 35.5-KeV γ -rays, as well as 22.1-KeV and 25.2-KeV fluorescence X-rays that are emitted from the silver bar. The lead half-layer thickness of ^125^I is 0.025 mm; thus, a lead sheet 0.025 mm thick can block more than 99% of the rays.

Presently, these particles can be locally produced. In 2001, the Institute of Atomic Energy of China (reactor engineering research and design) produced a CIAE-6711–^125^I particle seal that successfully passed the national quality assurance processes and is now widely used in clinical settings.

### Physical characteristics of the ^125^I particle

1. ^125^I releases gamma radiation with an average energy of 28 KeV, making it a low-power radioisotope. Thus, it is able to penetrate local tissues with good curative effects and little injury. The treatment ratio can be increased to 1.0 - 1.5 and 1.2 - 1.2.
2. The half-life of ^125^I is approximately 60 days; thus, its use can provide 200 days of continuous irradiation (three half-lives). The radiation emitted by ^125^I can extensively damage and break double-stranded DNA. The biological effects of ^125^I-generated radiation are appropriate for clinical use.
3. Because the lead half-value layer of ^125^I is 0.003 cm, it is easy to provide complete protection for the operator.
4. ^125^I is rapidly attenuated within the target treatment volume. Because the ^125^I radioactive nuclide is of low energy, *in vivo* tissue penetration is minimal (approximately 17 mm). It therefore is easy to protect surrounding tissues and organs, as the radioactive energy emitted by this isotope is not sufficient to damage the surrounding vital organs [21].
5. ^125^I provides a highly conformal dose distribution and is associated with a reduced incidence of late-responding tissue damage [22].

### ^125^I seed implantation techniques

The radioactive particles can be implanted temporarily or permanently. Currently, the ^125^I particles for clinical use are generally implanted permanently, meaning that particles are placed in tissues or lesions and are not later removed. The implantation methods used include percutaneous puncture implantation, stitching, adhesive fixation, tumour bed and draining area implantation, minimally invasive implantation, and cavity mirror implantation.

### Detection of the activity of ^125^I seeds

To control the quality of the particle source used for interstitial brachytherapy implants and to prevent medical exposure incidents, certain standard procedures must be followed. In accordance with Technical Report No. 1274 of the International Atomic Energy Agency, Calibration of Photon and Beta Ray Sources Used in Brachytherapy: Guidelines on Standardized Procedure sat Secondary Standards Dosimetry Laboratories (SSDLs) and Hospitals, 10% of the radioactive sources in each batch should be measured, and the activity of the measured particle sources should be maintained within 5% of the manufacturers’ values.

The ^125^I seed source air kerma rate can be measured using a well-type ionization chamber. This method is simple, quick, and can be used to estimate the apparent particle source activity [23].

### ^125^I particle dose calculation

The Memorial Sloan-Kettering Cancer Center was the first medical institution to use ^125^I seeds as an alternative radioactive source to ^222^Rn for permanent interstitial seed implants. The ^125^I photons are of low average energy and are attenuated to a much greater extent in tissue than higher-energy rays.

Currently, there is no uniform standard for the radiation dose of implanted ^125^I seeds in interstitial brachytherapy. Thus, we refer to the recommended standard proposed by the American Brachytherapy Society (2001) [24]: low dose rate after-loading irradiation should be administered at 40 to 45 Gy / 4 to 6 d at a dose rate of approximately 0.45 Gy/h (0.35 to 0.60 Gy/h). It is generally believed that a total dose of greater than 45 Gy significantly increases the incidence of complications [25].

Currently, treatment plans can be developed using computerized planning software. The operator need only enter the position and the image, and the treatment planning system (TPS) will automatically complete the design.

### Use of a TPS in ^125^I seed interstitial brachytherapy

The indicators used to evaluate a radiation therapy treatment plan are high tumour control probability and low normal tissue complication probability. The goal of such therapy is to irradiate a sufficiently large area of the tumour over at a sufficiently precise dose, while minimizing the radiation dose to surrounding normal tissues and reducing the radiation dose to surrounding normal tissue. The radiation dose can be calculated using TPS software, and an exact treatment plan can be formulated in the clinical setting. The main functions of the TPS software system are as follows:

1. Calculation of different radiation isodose curves (different tumours require different radiation doses).
2. Calculation of the position of the particle and the safe distance to radiation-sensitive tissues.
3. Calculation of the dose distribution range for minimally invasive treatment of subclinical lesions.
4. Calculation of parameters relevant to surgical applications (palliative resection and areas that may benefit from partial resection). Based on CT/MRI images, the target area is drawn on the TPS, and the numbers of implanted particles and required needles are calculated. There are two ways to implement a TPS: the Paris System Act and the peripheral dense, sparse Law Center. The former is used to determine the treatment volume (target) and to implant the radioactive particles in a uniform manner. The latter is used in treatments involving more uniform dose volumes.

## Clinical application of PVPI

i) For a successful PVP, it is key that experienced orthopaedic doctors or interventional radiologists have access to accurate real-time images. The most commonly used imaging equipment and technologies include X-ray machines (including the C-arm and the G-arm), CT, digital subtraction angiography (DSA), and intraoperative navigation system. G-arm, CT, and DSA can provide two-dimensional imaging, and the intraoperative navigation system can obtain three-dimensional images, all of which help physicians make smooth and precise adjustments during surgery and can significantly shorten the operative time.
ii) Preoperative preparation: Preoperative X-rays, bone scan images, CT, or MRI are used to determine the location and the number of affected vertebrae, the degree of vertebral collapse, the extent of osteolysis, the overall degree of damage, the integrity of the posterior wall of the vertebral body, pedicle violations, and the degree of spinal cord compression. Routine examinations such as tests of cardiopulmonary function, blood glucose, prothrombin time, liver and kidney function tests, iodine allergy test, and other parameters are also conducted.
iii) Surgical approach: For the cervical spine, the anterolateral or oral approach is used [26]. For the thoracolumbar spine, the pedicle approach is used. Sacral vertebrae require a direct near-surface approach.
iv) Needle position: The tip of the needle should be placed 1/3 of the way between the spinous process and the transverse process of the vertebra. The tip should be located within the pedicle shadow of the “bull’s-eye” sign when the needle reaches the cortical bone, but the depth should not exceed the leading edge of the pedicle. The tip is located in the upper or lower half of the vertebral body and should be directed towards one side. The centre of the vertebral body should be avoided in order to spare the vertebral central venous supply.
v) Implantation: When the needle enters the vertebral body, the needle core is removed. Using the needle as a channel, the implantation needle for the ^125^I seed is inserted into the needle tube. For the needle insertion process, the bilateral pedicle needle approach, in which one side of the needle tip is directed towards the upper vertebral body and the other side of the needle tip is directed towards the lower vertebrae, should be used if possible. Spacing of 0.3 cm around the target area of ^125^I seed implantation should be ensured, and the ^125^I seeds should be distributed three-dimensionally in the vertebral body. A bevelled tip is recommended to carry the puncture needle in the needle tip. The direction of the needle tip can be constantly adjusted as needed to permit implantation of ^125^I seeds in the ideal location.
vi) Contrast agent injection: Following puncture and ^125^I seed implantation, a contrast agent is injected at a volume of 5 mL. The diffusion of contrast agent and venous return is recorded by DSA. Depression suction for contrast agents and blood left in the vertebral body is used to reduce the pressure inside.
vii) Bone cement injection: After the bone cement is mixed, it is aspirated into the syringe and injected. Throughout the procedure, lateral fluoroscopy should be used to monitor the injection in order to determine if leakage of bone cement has occurred and to ensure that the cement-loaded needle is oriented in the proper direction. When the injection has been completed, the needle should be retracted to the cortical bone; the needle core is inserted, and the needle is rotated and removed to avoid sticking of the needle in the bone cement.
viii) Suitable period for bone cement injection: It is safe to inject bone cement while it is of “toothpaste” consistency.
ix) Volume of injected bone cement: The injection volume is generally 2 to 9 mL. The average volumes required are 2.5 mL in the cervical vertebral bodies, 5.5 mL in the thoracic vertebral bodies, and 7.0 mL in the lumbar vertebral bodies.

### Indications and contraindications for surgery

#### Indications

i) Patients who have the following characteristics: a clear medical history of cancer; a high suspicion of spinal metastases based on imaging; severe pain from vertebral collapse caused by metastases; and a requirement for bed rest and analgesics to relieve pain.
ii) Patients with decreased stability of the spine caused by metastases.
iii) Unexplained vertebral destruction that is surgically proven to be due to the presence of a primary tumour or metastatic spinal tumours.
iv) Patients with contraindications to open surgery or who are unwilling to undergo surgery.
v) As preliminary preparatory treatment for surgery and internal fixation, preoperative PVP can increase vertebral strength, partially embolize arteries, improve local conditions to reduce blood loss, and improve surgical safety Phase II [27,28].
vi) Patients whose estimated survival time is more than 2 months.

#### Contraindications

i) Patients with coagulation disorders or bleeding diathesis.
ii) Patients with severe peripheral osteolytic destruction, especially at the central border of the vertebral bodies. This is because leakage of bone cement or particle migration could damage the spinal cord or the adjacent blood vessels.
iii) Patients in poor physical condition, such as those with severe anaemia, cachexia, vital organ failure, or similar conditions.

### Complications and prevention

#### Leakage of PMMA

PMMA leakage is the most common complication, accounting for approximately 65% of cases [29]. It is closely associated with the filling amount; excessive injection of PMMA can cause increased pressure within the vertebral body. Bone cement acts as a liquid during perfusion. If the injection volume is increased blindly, the risk of PMMA leakage and compression fracture of the vertebral body increases. There are four types of bone cement spillover: type I (paraspinal), type II (intervertebral disc), type III (spinal canal), and type IV (mixed). In most cases, PMMA leakage is asymptomatic. However, if leakage reaches the spinal canal and foraminae, which are critical areas in an enclosed space, nerve damage can result. The complication rate is 5% to 8%; 3% to 6% of these patients have short-term symptoms of nerve root injury and require steroids or other anti-inflammatory drugs to relieve pain, and another 2% to 3% of cases require surgical decompression [30]. The amount of PMMA injected should be appropriately reduced in patients who have cortical destruction of the posterior and lateral edges, which can reduce the leakage risk of PMMA. Complete dural can effectively block leakage and prevent compression damage to the spinal cord [31].

Kyphoplasty can restore the compressed vertebral body height and correct vertebral kyphosis, and is a good choice for the treatment of vertebral osteoporotic compression fractures. However, compression fractures in spinal metastases are mainly accompanied by posterior edge defects. Posterior edge defects may worsen following the balloon dilatation that occurs during kyphoplasty, and the leakage rate may increase [32]. In addition, tumours within the vertebral body can be squeezed into the spinal canal during kyphoplasty balloon dilatation. Thus, we do not advocate the use of kyphoplasty for the treatment of vertebral metastases.

#### Pulmonary artery embolism and deep vein thrombosis

The complications of pulmonary artery embolism and deep vein thrombosis are mainly related to the entrance of fat or bone marrow into the venous circulation during the procedure. This phenomenon can be caused by venous diffusion of bone cement, especially if a needle is within the vertebral body communicates with a vein, which can occur when bone cement is released in an overly rapid injection. Bone cement aggregates generate heat, which can injure the vascular endothelium. Furthermore, monomers that enter the bloodstream can activate the complement system, increasing pulmonary vascular permeability and leading to activation of clotting factors. A hypercoagulable state develops, and fat and bone marrow tissue are released into the blood circulation, promoting thrombosis and leading in combination to pulmonary embolism. Chemical toxicity of the bone cement monomer promotes the release of mononuclear cells, which can cause deformation and separation of endothelial cells, resulting in the release of fibrous protein and the formation of pulmonary hypertension and blood clots [33]. Before injecting PMMA, vertebral angiography should be performed during the ‘toothpaste stage’, and high-pressure, rapid injections should be avoided.

#### Central vascular response

A central vascular response to PMMA is commonly seen in clinical settings. Most scholars believe that the bone cement monomer used in PMMA polymerization can inhibit myocardial contraction, resulting in low cardiac output and arrhythmias. However, some scholars believe that the bone cement monomer is not the direct cause of the decrease in cardiac output [34].

#### Adjacent vertebral fractures

In an *in vitro* biomechanical study, Ferguson [35] found that vertebroplasty may reduce the probability of segmental fracture but increased the probability of adjacent segmental vertebral compression fracture. The risk of fracture was related to the distribution of cement in the vertebral body and to the leakage of bone cement [36].

#### Myelitis

Both PVP surgery and ^125^I seed implantation can lead to myelitis. Myelitis induced by PVP alone is extremely rare, as it is a chemical or immunological reaction. However, ^125^I seed implantation-induced myelitis due to radiation damage, which causes multifactorial neuronal degeneration and necrosis. This complication is rare in clinical practice and requires further evaluation.

Yang et al. reported that irradiation with ^125^I triggered autophagy in neural cells; this autophagy stressed the endoplasmic reticulum and was primarily dependent on the PERK-eIF2α pathway [37]. In its early stages, the autophagy caused by ^125^I radiation may represent an attempt to increase cell survival, but it is a self-destructive process and ultimately promotes apoptosis and necrosis, which occur when cells are irradiated with ^125^I for more than 72 hours. Interference with PERK expression by intrathecal administration of a lentiviral vector can effectively inhibit autophagy and alleviate radiation myelitis in Banna pigs [37].

It has been reported in animal models that radiation myelitis due to ^125^I-based brachytherapy is related to the dose and duration of exposure. The rates of apoptosis and necrosis observed in spinal cord cells were effectively reduced with low doses of radiation and short treatment durations. At the dose prescribed in TPS, PVP in combination with ^125^I seed implantation has not been reported to induce radiation myelitis [38].

#### Tissue necrosis due to overdose of local irradiation

It has been reported that nearly 10% of patients require surgical procedures to remove necrotic tissue from surrounding brain areas following ^125^I-based brachytherapy for cerebral gliomas [39]. A similar situation has also been observed in prostate cancer therapy, where toxicity can occur in the urethra and rectum [40]. Therefore, radiation-induced damage to the adjacent spinal cord tissue should be given special consideration during preoperative treatment planning and postoperative follow-up.

#### Local pain

Local inflammation can be induced by local mechanical stimulation at the puncture site and by the heat that is produced during PMMA polymerization. A few hours after injection, transient exacerbation of pain or fever may occur; however, these symptoms are often alleviated in 2 to 4 days by treatment with anti-inflammatory drugs.

Other complications, such as infection, puncture damage to surrounding organs, and tumour seeding along the needle channel, are also possible.

### Effectiveness and mechanisms

Studies of the mechanism of PVP alone have generally focused on the study of its analgesic effects. However, in addition to its analgesic effects, PVP combined with ^125^I seed implantation can be an effective antitumour therapy.

### Dual mechanism of analgesia

#### Pure PVP treatment of spinal disorders can deliver a good analgesic effect

Overall, 90% of patients treated with PVP achieved pain relief within 6 to 72 h (mean 36 h); in some cases, rapid recovery has been observed [41,42]. In a previous study, the degree of pain relief was significantly greater in the treated group than in the control group after 2 months [43]. Nevertheless, the analgesic effect of PVP and the volume of bone cement injected are not positively correlated. For the treatment of vertebral metastases, a bone cement injection volume of 1.5 mL is sufficient to achieve a good analgesic effect [44]. However, the mechanism of action is not fully understood. Recent research has identified the following key mechanistic observations:

i) The polymerization of PMMA bone cement generates heat, and the PMMA monomer has a similar effect as absolute alcohol, which can cause the degeneration and necrosis of pain nerve fibres in vertebral bodies, leading to the loss of sensory function.
ii) Very small fractures can be repaired using bone cement, thereby eliminating stimulation of the nociceptive nerve endings due to extrusion and friction.
iii) After fixation with bone cement, improvements in spinal stability and a decrease in spinal stress may also help to relieve pain.
iv) Collapse of the tumour focus can reduce tension and widen the spinal canal and can decrease the pressure on surrounding tissues, increasing the pain receptor threshold.
v) Chemical media release the pain, which can be modulated. Pain can be observed to ease within a few days, which is mainly attributable to the decreased concentration of various chemical media. However, durable pain relief may be due to a reduced tumour burden and to calcification [45].

#### The mechanism of pain relief by ^125^I radioactive particles

Radiation therapy is directed at vertebral metastatic sites that are painful or are associated with significant epidural involvement. ^125^I radioactive particles emit low-energy γ -rays, thereby producing a sustained effect on tumour cells. Studies of interstitially implanted ^125^I radioactive particles in cancer treatment have demonstrated that pain significantly decreases approximately 5 to 7 days following implantation, improving patient quality of life.^125^I treatment may affect cancer pain through the following mechanisms:

i. Direct invasion of bone-activated local nociceptors by the tumour can compress neighbouring nerve blood vessels and soft tissues, causing pain. When the tumour invades the nerve root, sharp pain is produced in the nerve’s distribution area. Radiation can block conduction at peripheral nerve endings or affect the nerve sheath, leading to electrophysiological anaesthesia and stopping the pain pathway.
ii. Direct damage to nervous tissue. A day after tissue damage caused by irradiation, low electron density, granular degeneration, swelling of mitochondria, and visible shaft damage were observed by electron microscopy, leading to axoplasmic clearance of cellular material, which formed lacunae, altered the membrane axis, and caused separation of the myelin [46]. Within 10 days, the myelin sheaths lost resolution, and free myelin bubbles were visible with the nerve sheath, but myelin resolution was not obvious. Subsequently, changes were also seen in the sparse nerve fibres, with nerve fibre shrinkage and irregularity. The myelin collapsed and curled into irregular forms, with visible myelin degeneration and disintegration of residual nuclei. A study of these indirect injuries found that at 1 month after radiotherapy, endothelial cells showed inflammatory necrosis and depigmentation; repair led to vascular hyperplasia, leading to stenosis of the vascular lumen stenosis, thrombosis, and consequent nerve ischaemia. Marx and Johnson [47] showed that blood perfusion decreased in the irradiated area, reducing oxygen tension and leading to decreased oxygen saturation. In the irradiated area, the blood capillary density decreased by 20% to 30%, compared with the non-irradiated area. Ischaemia, hypoxia, acidosis and increased oxygen free radicals can affect various enzyme systems, altering cell metabolism and resulting in cellular edema, swollen mitochondria, metabolic disorders, and inhibition of protein synthesis. Lysosomal damage results in the release of proteolytic enzymes that destroy the tissue. Also affected peanut four dilute acid metabolism, reduce the synthesis of the top ring element, leading to an imbalance between the body top ring and thromboxane A2. Platelet aggregation and blood vessel damage are associated with hypercoagulability, further damaging the microcirculation; this process leads to nerve ischaemia and hypoxia and creates a vicious cycle. In the radiated area around the nerve, degeneration of nerve fibres, damage to axons, and myelin damage can be found. Near irradiated peripheral nerves, normal structures may be maintained; however, at more distant areas, a wide range of fibrotic contractures and scarring can occur gradually. These lesions can organize and lead to extensive stenosis and compression of the nerve fibres. If these changes continue, the neural microcirculation may be damaged, both internally and externally, and nerve transmission can be impaired.
  i) PG1 and PG2 can cause severe pain by bony destruction. Radiation can eventually kill cancer cells, reduce or stop the release of serotonin, and slow the release of pain factors such as kinin or prostaglandin.
  ii) The tumour may infiltrate blood vessels and lymphatic vessels, which stimulate the nerves that surround blood vessels. Vascular occlusion can lead to tissue oedema, fascial disruption, and other consequences, all of which can produce severe pain. If malignant tumours are damaged by radiation-induced microthrombus formation or fibrosis, the production of pain factors can be completely blocked.

In conclusion, it is thought that after ^125^I radioactive particle implantation, pain caused by malignancies is relieved primarily by inhibition of the release of pain factors and blocking of nerve impulse transmissions.

### The mechanism of PMMA and ^125^I in spinal metastases

#### Dual mechanism of tumour destruction

The mechanism though which PMMA kills tumour cells remains unclear. Nevertheless, the following potential mechanisms have been proposed:

i) During polymerization of the PMMA resin bone cement, a large amount of heat is generated; this could effectively inactivate the tumour cells around the bone cement [48], which are poorly heat-resistant and may undergo necrosis. The production of inflammatory and pain mediators would decrease, the growth of tumour cells would be prevented, and the compression of nerve endings would decrease. However, under these conditions, normal tissues experience no irreversible damage.
ii) Bone cement can infiltrate tumour tissues and solidify, thereby separating part of the tumour tissue from its feeding vessels, leading to necrosis of tumour cells [48].
iii) The bone cement monomer has a similar effect on tissues as absolute alcohol, which can cause cell dehydration, solidification, and death [48].

The mechanism and effects of the killing of tumour cells by bone cement need to be further studied. The usefulness of this effect is limited to the areas surrounding the bone cement; some tumour cells may still survive and continue to invade adjacent tissues, decreasing the long-term efficacy of PVP treatment. Thus, PVP combined with ^125^I radioactive particle implantation effectively kills tumour cells.

### Tumouricidal mechanism of ^125^I seeds

There are many ways to kill tumour cells by ionizing radiation. In addition to the direct killing effect, an important mechanism of radiotherapy is the induction of tumour cell apoptosis. Compared to all previous radiation treatments, permanent interstitial implantation of ^125^I particles provides continuous irradiation at a lower dose rate [49]. Many researchers have studied the effects on cells of a low dose rate and single irradiation; these studies demonstrated that, within a certain dose rate range, cells were radiosensitized at a lower dose rate. The sensitivity of cells to radiation is a comprehensive reflection of a variety of mechanisms, including radiation-induced apoptosis, which is an important factor [50-52]. The cell cycle was blocked when tissues were irradiated with ^125^I seeds. The number of cells in the G2 and S phases increased, and cell cycle redistribution occurred. As cells in the G2 and S phase are more sensitive to γ rays [53], the killing effects on tumour cells caused by ^125^I particle irradiation would be expected to increase.

The low-energy radiation released by ^125^I seeds consists primarily of γ rays, which can damage the nuclear DNA of tumour cells, inducing tumour cell apoptosis and causing them to lose the ability to divide.

Radioactive implants in tumours mainly interact with substances via the photoelectric effect, the Compton effect, and electron pair effects, all of which cause changes in molecular structure and function. As a result, the RNA and DNA content of the cells decreases, catabolite levels increase, and the intracellular environment is damaged, resulting in imbalance of the regulation of activities at a cellular level. Finally, apoptosis of tumour cells occurs [54].

i) Direct effect: Ionizing radiation has a direct effect on biological macromolecules, which compose cells. Energy from ionizing radiation is deposited directly in biological macromolecules by the direct effect of radiation. When this occurs, biological macromolecules are ionized, and the material, including nucleic acids, proteins, enzymes, etc., is destroyed. Only a small fraction of cells (called active phase cells) undergo continuous reproduction during tumour growth. The cell cycle is divided into four phases: the phase prior to DNA synthesis (G1 phase), the DNA synthesis phase (S phase), the phase following DNA synthesis (G2 phase), and mitosis (M phase). Exposure of cells in the late DNA synthesis and mitosis phases to only a small amount of γ rays (3 cGy) can destroy the nuclear DNA of tumours, which lose their ability to reproduce and proliferate.
ii) Indirect effects: ionizing radiation acts directly on water molecules, decomposing them into a series of products such as H^+^, OH^-^, HO^-2^, H_2_O_2_ and other free radicals. These decomposition products then act on biological macromolecules, altering their physical and chemical characteristics [55]. These two types of effects often coexist in living cells, and they complement each other. Free radicals display high reactivity, instability, and paramagnetism and have the following effects on biological molecules:

1. Free radicals participate in chemical reactions, including addition reactions, electron capture reactions, disproportionation reactions, reduction reactions, and oxidation reactions, among others.
2. Free radicals can damage DNA. For example, pyrimidines and purines can be damaged by the addition reaction caused by free radicals.
3. Peroxidation of lipids, in which oxygen radicals attack polyunsaturated fatty acids in membrane phospholipids and lipid hydroperoxides are produced by lipid peroxidation, can occur. The role of active oxygen is amplified by chain and branched-chain reactions, and active oxygen can decompose many lipids. Some of the decomposition products can induce barriers to cell metabolism and function, leading to cell damage.

The mechanisms of these effects may include changes in membrane lipids, changes in membrane function, and damage to membrane enzymes, as well as cytotoxic effects of the decomposition products of lipid peroxidation.

The spatial structure of membrane proteins is changed by the exposure of cells to continuous low-dose radiation, and the ion channel activity of the membrane decreases or becomes inactive. Generally, endothelial cell proliferation is blocked, and the transmission of information between cells and the supply of nutrients to the surrounding tissues is reduced. All these effects lead to tumour cell apoptosis.

## The advantages and prospects of PVPI for the treatment of bone metastases

Yang et al. successfully established an animal model of PVPI in miniature pigs, and the safety of minimally invasive surgery has thus been verified [56,57]. PVP is a minimally invasive procedure that is associated with less trauma, simple operations, and usually only minor complications. It can effectively alleviate pain in patients with osteolytic spinal metastases. The antitumour effects of PVP can be enhanced if it is performed in combination with interstitial implantation of ^125^I seeds [5]. Further studies have shown that spinal osteoblastic metastases are not a contraindication for PVP surgery. In PVP surgery, bone cement can be successfully injected and distributed in the peripheral or central regions of osteoblastic lesions. Mechanical strength is improved, pain is controlled, and the growth of local tumour lesions is inhibited. PVP is more effective when it is combined with ^125^I seed implantation [5].

Compared with other existing treatments, PVP combined with ^125^I seed implantation to treat spinal metastases has the following advantages:

1. Only low-energy radiation is released by ^125^I particles. Most radiation is absorbed by the tumour tissues; a very conformal high-dose area can be accurately designed within the tumor, allowing the continuous delivery of radiotherapy. Thus, γ rays can continuously kill tumour cells, whereas there is limited destruction to the surrounding normal tissues.
2. The mechanical strength of the vertebral body is increased, while continuous irradiation at a low dose rate inhibits the mitosis of tumour cells. Tumour cells accumulate in the G2 phase, and tumour cell proliferation is significantly reduced. Tumour foci become stagnant and are destroyed by multiple mechanisms. The tumour volume is reduced, and the condition of local tissues is improved. PVP can be used before surgery to avoid compression fractures of vertebral bodies during preparation for surgery [58,59].
3. Minimally invasive surgical techniques are used for implant therapy, and the procedure is associated with shorter operative times, shorter hospital stays, little pain, and rapid postoperative recovery. Because its clinical efficacy is clear, it is easily accepted by patients; furthermore, postoperative patient quality of life is significantly improved.
4. Simple equipment is used in the treatment, which has low costs.
5. Compared with interstitial ^125^I brachytherapy only, the use of bone cement can fix defects and theoretically reduce the possibility of particle removal.
6. The mechanical properties and efficacy of bone cement are not affected by radiation [16,60,61].

Lu et al. reported that local tumours still progressed during follow-up after treatment with PMMA loaded with antineoplastic drugs, suggesting that chemotherapy drugs do not completely inhibit tumour growth. Vertebroplasty combined with external radiotherapy can enhance local anti-tumour effects, but this approach requires approximately 3 weeks of external beam radiotherapy and affects the total treatment time for the primary lesion [62,63]. Yang et al. reported that PVPI had considerable local anti-tumour activity and that comprehensive treatment of patients, including chemotherapy, for 1 week can significantly enhance overall outcomes [63].

## Conclusions

In conclusion, PVPI is a safe, reliable, effective, and minimally invasive therapy. PVP and ^125^I seed implantation enhance each other’s effects and represent a novel, broadly applicable method of treating spinal metastases.

Currently, PVPI technology is still exploratory, and further research is required; a large amount of clinical and basic research is required. Future research should focus on possible complications and sequelae, methods of using TPS to design and precisely control the spacing of implanted particles, methods of optimally distributing the particle sources in accordance with the target tissue volume and density, as well as the relationship between adjacent vital organs, and how to achieve “directional blasting” to maximize the killing of tumour cells and minimize damage to normal tissue and functions.

## Abbreviations

^125^I: Iodine-125
DSA: Digital subtraction angiography
PMMA: Polymethylmethacrylate
PVP: Percutaneous vertebroplasty
PVPI: PVP and ^125^I seed implantation
